# GOLPH3: a novel biomarker that correlates with poor survival and resistance to chemotherapy in breast cancer

**DOI:** 10.18632/oncotarget.21927

**Published:** 2017-10-19

**Authors:** Shicong Tang, Hong Pan, Wei Wei, Huawei Yang, Jianlun Liu, Rirong Yang

**Affiliations:** ^1^ Department of Breast Surgery, Affiliated Tumor Hospital of Guangxi Medical University, Nanning, Guangxi, People's Republic of China; ^2^ Department of Thoracic Surgery, Affiliated Tumor Hospital of Guangxi Medical University, Nanning, Guangxi, People's Republic of China; ^3^ Department of Immunology, School of Preclinical Medicine, Guangxi Medical University, Guangxi, People's Republic of China

**Keywords:** GOLPH3, breast cancer, chemotherapy, survival

## Abstract

The association between Golgi phosphoprotein 3 (GOLPH3) and clinical pathological characteristics, as well as the clinical outcomes of both neoadjuvant and adjuvant chemotherapies in breast cancer, remain largely unknown. In this study, we investigated the biological role and clinical significance of GOLPH3 in breast cancer. We found that GOLPH3 expression in tumor tissue was higher than that in adjacent noncancerous tissue (ANT) and fibroadenoma. GOLPH3 silencing reduced the migration, invasion, and proliferation of breast cancer cells and promoted apoptosis of the cells. Importantly, patients with high GOLPH3 expression had worse disease-free survival (DFS) and overall survival (OS), and GOLPH3 expression was correlated with clinical pathological characteristics such as molecular subtype, tumor-node-metastasis classification, and age but was not associated with surgery type. Patients with high GOLPH3 expression had poor DFS and OS in every molecular subtype, and an increase in tumor invasion and lymph node metastasis. The risk of recurrence increased with age in patients with high GOLPH3 expression, and surgery type had no influence on patient survival. This is the first study to investigate the correlation between GOLPH3 and response to chemotherapy in breast cancer. Patients with high GOLPH3 expression showed resistance to neoadjuvant and adjuvant chemotherapies, and GOLPH3 overexpression indicated a high risk of recurrence in patients who received adjuvant chemotherapy. These data suggest that GOLPH3 may be a novel biomarker that correlates with poor survival and resistance to chemotherapy in breast cancer.

## INTRODUCTION

Breast cancer is the most common malignant tumor in females worldwide [1–3], and its incidence is increasing [4]. Tumor invasion and metastasis are the major causes of treatment failure in this disease; thus, it is important to discover novel molecular biomarkers that can serve as targets for its diagnosis and treatment.

Golgi phosphoprotein 3 (GOLPH3, also named GMx33 or GPP34) and its interacting proteins are hot topics in cancer research [5–7]. GOLPH3 is a highly conserved 34-kDa trans-Golgi matrix membrane protein that plays a key role in receptor recycling, glycosylation, and protein trafficking from Golgi to the plasma membrane [8–11]. Its expression is associated with tumor migration and proliferation in some cancers such as bladder, lung, ovarian epithelial, prostate, liver, rectal, and kidney cancers [18, 20–25]. It is suggested that GOLPH3 functions as an oncogene in tumorigenesis and migration [12–15] by activating the mechanistic target of rapamycin (mTOR), Wnt, and nuclear factor kappa-light-chain-enhancer of activated B cells (NF-kB) signaling pathways and by upregulating matrix metalloproteinases (MMPs) such as MMP-2 and MMP-9 [16–19]. Zeng *et al.* [26] found that GOLPH3 is highly expressed in breast cancer, but its function needs further investigation.

Some reports have shown that higher levels of GOLPH3 are associated with a worse prognosis as determined by disease-free survival (DFS) or overall survival (OS). For example, high GOLPH3 overexpression correlates with a worse DFS in small cell lung cancer [18][23], prostate cancer [21], ovarian epithelial cancer [20], hepatoma carcinoma [22], and rectal carcinoma [25]. It is also correlated with poor OS in renal cell carcinoma [24] and breast cancer [26]. However, little is known about the role that GOLPH3 plays in patients’ response to breast cancer treatment. Thus, additional studies are needed to explore the relationship between GOLPH3 expression and clinical outcomes and response to therapy.

In this study, we analyzed the expression of GOLPH3 in breast cancer and its effects on migration and proliferation in breast cancer cell lines. Then we investigated the association between GOLPH3 expression and clinical characteristics to determine if GOLPH3 expression affects recurrence, OS, and the response to neoadjuvant chemotherapy.

## RESULTS

### GOLPH3 is highly expressed in breast cancer tissue and cell lines

**Figure 1:**
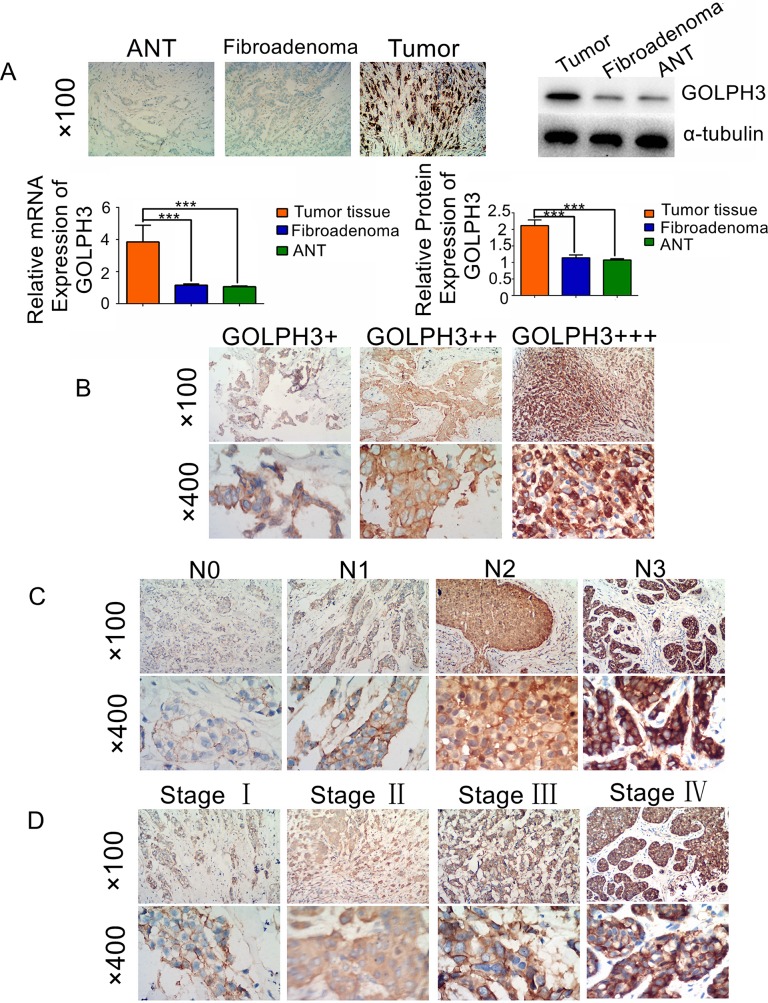
GOLPH3 is overexpressed in breast cancer **(A)** GOLPH3 expression in breast cancer tissue is higher than that in adjacent noncancerous tissues (ANTs) and fibroadenoma, as determined by immunohistochemistry, Western blotting, and real-time PCR. **(B)** GOLPH3 expression was demonstrated by immunohistochemistry. **(C)** GOLPH3 protein expression in patients with different grades of lymph node metastasis was detected by immunohistochemistry. **(D)** GOLPH3 protein expression in patients at different clinical stages was detected by immunohistochemistry. All values are shown as the mean ±SD of three independent experiments. ^***^, *p*<0.001.

To assess GOLPH3 expression in tumor tissues, adjacent noncancerous tissue (ANT), and fibroadenomas, quantitative PCR (qPCR), immunohistochemistry, and Western blotting were performed. Compared with ANT and fibroadenomas, there were significantly higher levels of GOLPH3 in breast cancer tissues and no significant difference in expression between ANT and fibroadenomas (Figure 1A). In addition to tissue samples, the expression of GOLPH3 in two types of breast cancer cell lines was examined: the MDA-MB-231 cell line, which has a high potential of invasiveness, had higher GOLPH3 expression than the MCF-7 cell line (Supplementary Figure 1A). The immunohistochemistry results showed that GOLPH3 expression levels could be classified into +, ++, and +++ (Figure 1B). Moreover, its expression increased as the tumor-node-metastasis (TNM) classification increased (Figures 1C, 1D).

### Silencing of GOLPH3 expression reduces cell migration, invasion, proliferation and promotes the apoptosis of breast cancer cells

**Figure 2:**
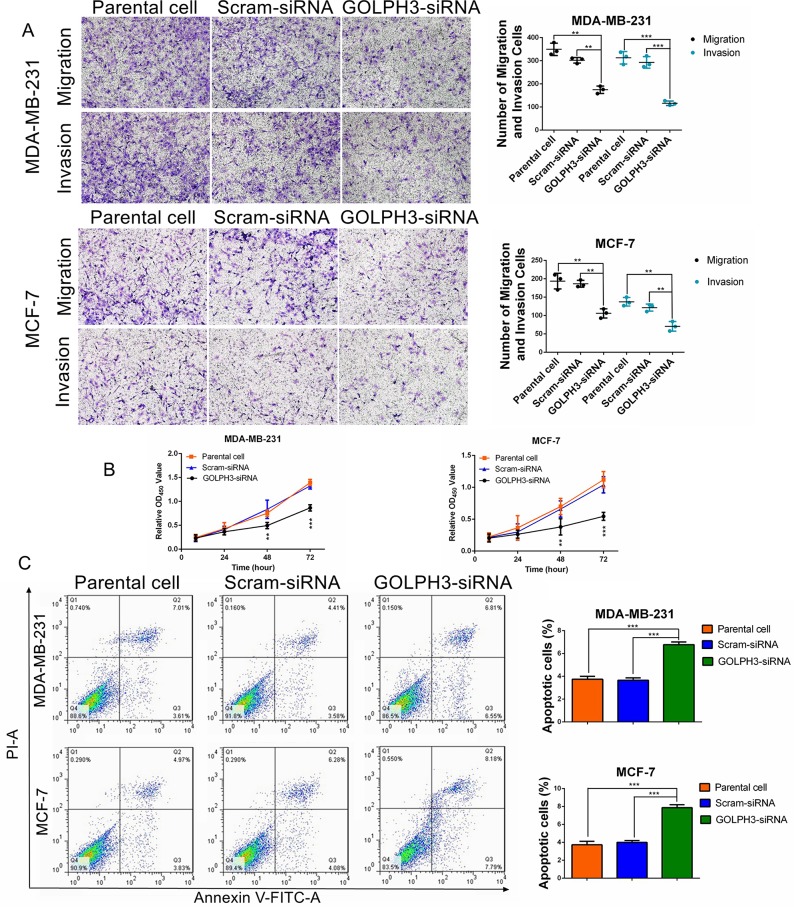
GOLPH3 silencing inhibits breast cancer cell migration, invasion, and proliferation, and promotes apoptosis **(A)** Downregulation of GOLPH3 reduced breast cancer cell migration and invasion. Magnification 100×. **(B)** GOLPH3 silencing suppressed the proliferation of both MDA-MB-231 and MCF-7 cells. **(C)** GOLPH3 silencing promoted the apoptosis of both MDA-MB-231 and MCF-7 cells. All values are the mean ±SD of three independent experiments. ^**^, *p*<0.01. ^***^, *p*<0.001.

To elucidate whether GOLPH3 affects cell migration, invasion, and proliferation, short interfering RNA (siRNA) was successfully transfected into the MCF-7 and MDA-MB-231 breast cancer cell lines. The downregulation of GOLPH3 was confirmed by qPCR and Western blotting (Supplementary Figure 1B). The migration and invasion of breast cancer cells were significantly reduced in cells transfected with GOLPH3-siRNA compared with parent cell lines and cells transfected with scram-siRNA (*p<*0.05) (Figure 2A). No significant difference was found between parent cell lines and cells transfected with scram-siRNA (Figure 2A). Cell proliferation assessed by the CCK-8 cell viability assay showed that cells transfected with GOLPH3-siRNA had reduced proliferation compared with parent cell lines and cells transfected with scram-siRNA (Figure 2B). These data indicate that silencing of GOLPH3 suppresses breast cancer cell migration, invasion, and proliferation. To investigate how GOLPH3 expression influences cell apoptosis, the Annexin V FITC apoptosis detection kit was used followed by flow cytometry. In both MDA-MB-231 and MCF-7 cell lines, there was a greater accumulation of early apoptotic cells in GOLPH3-siRNA cells compared with parental and scram-siRNA cells, indicating that GOLPH3 knockdown induced breast cancer cell apoptosis (Figure 2C).

### GOLPH3 overexpression was associated with worse survival in breast cancer patients

**Figure 3:**
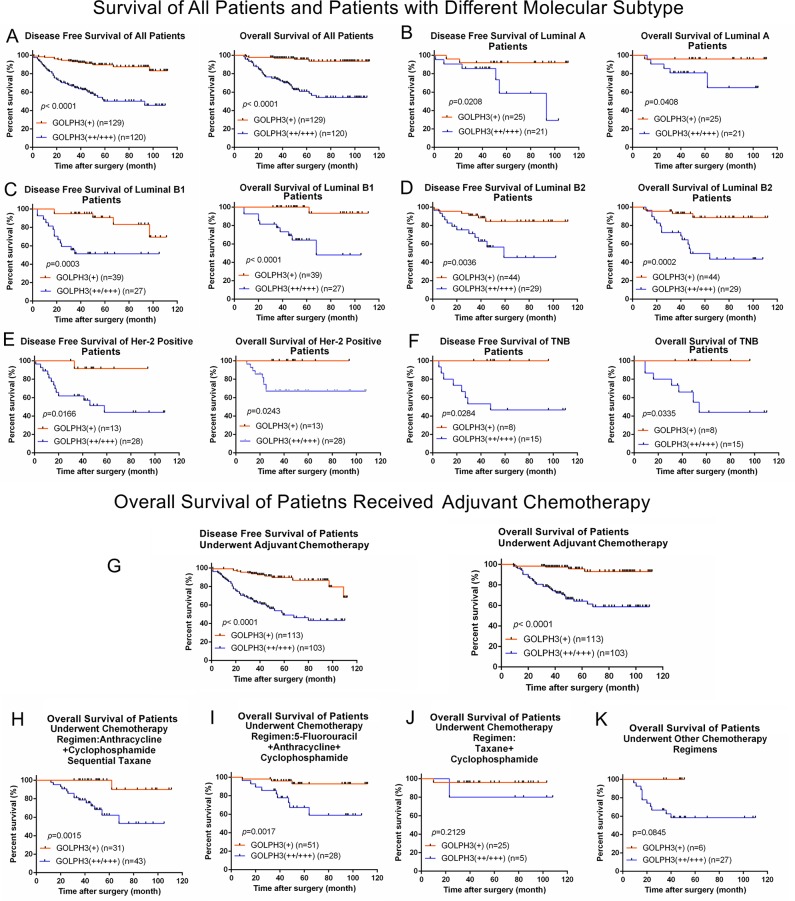
Disease-free survival and overall survival of breast cancer patients with different GOLPH3 expression levels **(A)** DFS and OS were analyzed by the Kaplan–Meier method for breast cancer patients with low GOLPH3 expression (GOLPH3+) (n=129) compared to those with high GOLPH3 expression (GOLPH3++/+++) (n =120). **(B)** DFS and OS were analyzed in patients with luminal A breast cancer. **(C)** DFS and OS were analyzed in patients with luminal B1 breast cancer. **(D)** DFS and OS were analyzed in patients with luminal B2 breast cancer. **(E)** DFS and OS were analyzed in patients with HER-2 positive breast cancer. **(F)** DFS and OS were analyzed in patients with TNB breast cancer. **(G)** DFS and OS of patients received adjuvant chemotherapy. **(H)** OS of patients treated with anthracycline+cyclophosphamide sequential taxane. **(I)** OS of patients treated with 5-fluorouracil+anthracycline+cyclophosphamide. **(J)** OS of patients treated with taxane+cyclophosphamide. **(K)** OS of patients treated with other chemotherapy regimens.

The clinical pathological characteristics of the 249 breast cancer patients are summarized in Table 1 and Supplementary Table 1, and no patients were lost during follow-up. We analyzed the correlation between GOLPH3 expression and age, T classification (tumor invasion depth), N classification (lymphatic metastasis), M classification (distant metastasis), molecular subtype, and surgery type. GOLPH3 expression was associated with T classification (p=0.033), N classification (p<0.001), M classification (p<0.001), molecular subtype (p=0.012), and surgery type (p<0.001). Compared with patients with low GOLPH3 expression, patients with high expression had worse DFS and OS (Figure 3A). Univariate analysis showed that T, N, and M classifications, molecular subtype, and GOLPH3 expression were associated with DFS and OS (Table 2), and multivariate analysis showed that these factors were important prognostic factors for the DFS of breast cancer patients (Table 3). Moreover, N classification, molecular subtype, and GOLPH3 overexpression were highly correlated with the OS of breast cancer patients (Table 3). Taken together, these data indicate that GOLPH3 is an independent prognostic factor for DFS and OS in breast cancer. Interestingly, we found that patients with GOLPH3 overexpression had poor DFS and OS in every molecular subtype. For example, in patients with luminal A breast cancer, high GOLPH3 expression correlated with poor DFS (p=0.0208) and OS (p=0.0408) (Figure 3B). Similar results were observed for both DFS and OS in other molecular subtypes (luminal B1, luminal B2, human epidermal growth factor receptor 2-positive, and triple-negative breast [TNB]; Figures 3C–3F). However, the difference in both DFS and OS across the five molecular subtypes was not significant (DFS: p=0.6979, OS: p=0.6527) (Supplementary Figures 2A, 2B). Similarly, in patients with low GOLPH3 expression, no significant differences in DFS and OS among the five molecular subtypes were found (DFS: p=0.7547, OS: p=0.4734) (Supplementary Figures 2A, 2B).

**Table 1:** Association between GOLPH3 expression and the clinicopathological features of 249 breast cancer patients

Variables	Total (n=249)	Expression of GOLPH3		P-value
		Low (n=129)	High (n=120)	
**Age**				0.080
<40	34	21	13	
40≤Age<50	90	37	53	
50≤Age<60	80	45	35	
≥60	45	26	19	
**T classification**				0.033
T1	21	16	5	
T2	183	95	88	
T3	28	13	15	
T4	17	5	12	
**N classification**				<0.001
N0	94	87	7	
N1	69	38	31	
N2	56	4	52	
N3	30	0	30	
**M classification**				<0.001
M0	238	129	109	
M1	11	0	11	
**Molecular subtype**				0.012
Luminal A	46	25	21	
Luminal B1	66	39	27	
Luminal B2	73	44	29	
Her-2 positive	41	13	28	
Triple negative	23	8	15	
**Surgery type**				<0.001
Simple mastectomy	44	41	3	
Modified radical mastectomy	179	68	111	
Breast conserving surgery	18	17	1	
Section resection	8	3	5	

Note. Luminal A: ER (+) and PR (+), PR≥20%, Her-2 (−), Ki-67<14%; Luminal B1: ER (+), Her-2 (−), Ki-67≥14% or PR<20%; Luminal B2: ER(+), Her-2 (+), Ki-67≥14% or PR<20%; Her-2 positive: ER (−) and PR (−), Her-2 (+); Triple negative: ER (−) and PR (−), Her-2 (−). The results was corrected to three decimal places.

**Table 2:** Kaplan-Meier survival analysis of GOLPH3 expression in breast cancer patients

Variables	Total (n=249)	DFS		P value	OS		P value
		Mean (month)	Median (month)		Mean (month)	Median (month)	
**Age**				0.145			0.228
<40	34	90.97	NA		89.73	NA	
40≤Age<50	90	84.28	NA		95.07	NA	
51≤Age<60	80	82.23	109.00		89.75	NA	
≥60	45	80.70	NA		79.05	NA	
**T classification**				0.000			0.023
T1	21	96.70	NA		98.55	NA	
T2	183	88.43	NA		93.84	NA	
T3	28	55.59	46.00		82.44	NA	
T4	17	36.60	40.00		43.42	NA	
**N classification**				0.000			0.000
N0	94	110.86	NA		109.82	NA	
N1	69	85.13	109.00		99.38	NA	
N2	56	55.87	52.00		72.30	68.00	
N3	30	35.14	30.00		41.30	35.00	
**M classification**				0.000			0.005
M0	238	85.43	NA		93.28	NA	
M1	11	34.80	40.00		49.64	NA	
**Molecular subtype**				0.046			0.029
Luminal A	46	94.51	NA		98.41	NA	
Luminal B1	66	84.70	NA		94.55	NA	
Luminal B2	73	86.98	NA		88.34	NA	
Her-2 positive	41	73.79	NA		88.13	NA	
Triple negative	23	78.57	NA		80.89	NA	
**Surgery type**				0.158			0.225
Simple mastectomy	44	99.39	NA		101.02	NA	
Modified radical mastectomy	179	76.88	109		87.99	NA	
Breast conserving surgery	18	106.21	NA		107.14	NA	
Section resection	8	70.00	NA		73.05	NA	
**GOLPH3 expression**				0.000			0.000
Low	129	101.61	NA		107.22	NA	
High	120	67.125	93.00		74.86	NA	

Note. Luminal A: ER (+) and PR (+), PR≥20%, Her-2 (−), Ki-67<14%; Luminal B1: ER (+), Her-2 (−), Ki-67≥14% or PR<20%; Luminal B2: ER (+), Her-2 (+), Ki-67≥14% or PR<20%; Her-2 positive: ER (−) and PR (−), Her-2 (+); Triple negative: ER (−) and PR (−), Her-2 (−). The results was corrected to three decimal places.

Abbreviations: DFS, disease free survival; OS, overall survival; NA, none detected.

**Table 3:** Cox multivariate analysis of disease free survival and overall survival

Variables	Disease free survival			Overall survival		
	Hazard ratio	95% confidence interval	P-value	Hazard ratio	95% confidence interval	P-value
**Age**	1.022	0.999-1.046	0.061	1.105	0.987-1.043	0.305
**T classification**	1.432	1.026-1.998	0.035	1.036	0.680-1.576	0.870
**N classification**	2.766	1.859-4.114	0.000	2.498	1.570-3.975	0.000
**M classification**	1.780	0.770-4.111	0.177	1.645	0.624-4.335	0.314
**Molecular subtype**	1.333	1.087-1.635	0.006	1.331	1.054-1.681	0.016
**Surgery type**	1.006	0.625-1.617	0.982	0.955	0.508-1.795	0.886
**GOLPH3 expression**	1.897	1.536-3.913	0.003	2.013	1.442-3.209	0.002

Note. The results was corrected to three decimal places.

### Association of age, TNM classification, and survival with GOLPH3 expression

Although the results shown in Table 1 indicated that there was no statistically significant difference in mean patient age between those with high and low GOLPH3 expression (p=0.080), we categorized age into four groups (<40, 40–50, 50–60, >60) to evaluate the effect of GOLPH3 expression on each group. In patients <40 years of age, those with GOLPH3 overexpression did not have an increased risk of breast cancer recurrence (p=0.5659), but their OS was significantly poorer (p=0.0187) (Supplementary Figure 2C). Interestingly, patients with high GOLPH3 expression in the other three age groups had poorer DFS and OS compared with those with low GOLPH3 expression (Supplementary Figures 2D–2F). There was no statistical significance in DFS and OS in patients with low GOLPH3 expression among the four age groups (Supplementary Figure 2G). Importantly, the risk of recurrence increased with age in patients with GOLPH3 overexpression (Supplementary Figure 2H); however, no significant difference in OS was observed in the four age groups (Supplementary Figure 2H). GOLPH3 expression was highly correlated with tumor invasion depth (p=0.033) (Table 1). We also assessed DFS and OS in patients with GOLPH3 expression as well as tumor invasion depth in patients with T1–T4 lesions. For patients with T1 or T2 lesions, high GOLPH3 expression led to poorer DFS and OS compared with those with low GOLPH3 expression (T1, DFS: p=0.0103; OS, p=0.0101; T2, DFS: p<0.0001; OS: p<0.0001) (Supplementary Figures 3A, 3B). However, in patients with T3 or T4 lesions, there was no significant difference in DFS or OS between patients with high or low GOLPH3 expression (T3, DFS: p=0.1389, OS: p=0.1270; T4, DFS: p=0.1203, OS: p=0.4716) (Supplementary Figures 3C, 3D). For patients with high GOLPH3 expression, T3 and T4 lesions indicated poor DFS (p=0.0360) but not poor OS (p=0.7521) (Supplementary Figure 3F). For patients with low GOLPH3 expression, an increase in tumor invasion depth correlated with worse DFS (p=0.0186) and OS (p=0.0222) (Supplementary Figure 3E). The relationship between GOLPH3 expression and lymphatic metastasis was also observed in our study (p<0.001) (Table 1). For patients without lymphatic metastasis (N0), high GOLPH3 expression indicated poor DFS (p=0.0004) and OS (p=0.0166) (Supplementary Figure 4A). However, for patients with lymphatic metastasis (N1 and N2), GOLPH3 expression appeared to have no influence on DFS and OS (N1, DFS: p=0.3957, OS: p=0.6825; N2, DFS: p=0.4373, OS: p=0.6775) (Supplementary Figures 4B, 4C). In patients with low GOLPH3 expression, the increase in lymphatic metastasis correlated with poorer DFS (p<0.0001) rather than OS (p=0.3053) (Supplementary Figure 4D). While in patients with high GOLPH3 expression, the increase in lymphatic metastasis led to a worse DFS (p=0.0012) and OS (p<0.0001) (Supplementary Figure 4E).

### Surgery type has no influence on the survival of patients with high GOLPH3 expression

Besides disease characteristics, we focused on the relationship between GOLPH3 expression and surgery type (breast conserving surgery, simple mastectomy, modified radical mastectomy, section resection) to find a suitable surgery type for patients with GOLPH3 overexpression. In the cohort of patients who received breast-conserving surgery, 17 had high GOLPH3 expression and 1 had low expression. Because of the small number of patients, no effects of GOLPH3 expression were found on DFS and OS in patients receiving breast-conserving surgery (Supplementary Figure 5A). The same results were observed in patients who received simple mastectomy and section resection (Supplementary Figures 5B, 5C). High GOLPH3 expression was associated with poorer DFS and OS in patients who received modified radical mastectomy, (p<0.0001) (Supplementary Figure 5D), whereas low expression had no effect on DFS and OS regardless of surgery type (Supplementary Figure 5E). In patients with high GOPH3 expression, the results of DFS and OS showed that there was a significant difference across the four surgery types (DFS: p=0.0240, OS: p=0.0182) (Supplementary Figure 5F). However, these results were based on data from a small number of patients; thus, future studies are needed with a larger cohort of patients to confirm these results.

### High GOLPH3 expression is correlated with neoadjuvant chemotherapy resistance in breast cancer patients

In our study, 54 patients received neoadjuvant chemotherapy. The partial response (PR) in 36 patients with high GOLPH3 expression was lower than that in the 18 patients with low expression (p=0.021) (Table 4). In this patient cohort, 22 patients received the anthracycline+taxane+cyclophosphamide regimen, 20 received the 5-fluorouracil+anthracycline+cyclophosphamide regimen, and 12 received other regimens. In the group treated with the anthracycline+taxane+cyclophosphamide regimen, the PR in the 14 patients with high GOLPH3 expression had lower PR rates (6/14) than the 8 patients with low GOLPH3 expression (p=0.05) (Table 5). In the group of patients who underwent 5-fluorouracil+anthracycline+cyclophosphamide and other regimens, the PR rates were not statistically different between high and low GOLPH3 expression (p=0.492 and p=0.296, respectively) (Table 5).

**Table 4:** Correlation between GOLPH3 expression and effect of neoadjuvant chemotherapy

GOLPH3 expression	Total (n)	PR (n)	SD (n)	P-value
GOLPH3 +	18	15	3	0.021
GOLPH3 ++/+++	36	18	18	
Total	54	33	21	

Abbreviations: PR, partial response; SD, stable disease.

**Table 5:** Correlation between GOLPH3 expression and effect of different neoadjuvant regimens

Neoadjuvant regimen	GOLPH3 expression	Total (n)	PR (n)	SD (n)	P-value
Anthracycline + taxane + cyclophosphamide (n=22)	GOLPH3 +	8	7	1	0.05
	GOLPH3 ++/+++	14	6	8	
5-fluorouracil + anthracycline + cyclophosphamide (n=20)	GOLPH3 +	9	7	2	0.492
	GOLPH3 ++/+++	11	7	4	
Others (n=12)	GOLPH3 +	1	1	0	0.296
	GOLPH3 ++/+++	11	5	6	

Abbreviations: PR, partial response; SD, stable disease.

### High GOLPH3 expression is correlated with high recurrence rate in breast cancer patients who received adjuvant chemotherapy

We also evaluated the relationship between GOLPH3 expression and breast cancer recurrence in patients who received adjuvant chemotherapy after surgery. A total of 216 patients received adjuvant chemotherapy after surgery. Patients with high GOLPH3 expression (n=103) showed poorer DFS and OS compared with those with low expression (n=113) (DFS: p<0.0001, OS: p<0.0001) (Figure 3G). Among the patients who received adjuvant chemotherapy, 74 received the anthracycline+cyclophosphamide sequential taxane regimen. Patients with high GOLPH3 expression had a higher risk of recurrence (p=0.035) (Table 6) and poor OS (p=0.0015) (Figure 3H). Among those who received adjuvant chemotherapy, 79 patients received 5-fluorouracil+anthracycline+cyclophosphamide. Patients with high GOLPH3 expression had the same risk of recurrence and death as those who received anthracycline+cyclophosphamide sequential taxane regimen (DFS: p=0.010, OS: p=0.0017) (Table 6 and Figure 3I). Of the 30 people who received taxane+cyclophosphamide regimen, no significant difference in recurrence or OS was observed between those with high and low GOLPH3 expression (DFS: p=0.190, OS: p=0.2129) (Table 6, Figure 3J). In patients who received other chemotherapy regimens, GOLPH3 overexpression indicated a high risk of recurrence (p=0.027) (Table 6) but no statistical difference in OS (p=0.0845) (Figure 3K).

**Table 6:** Recurrence between GOLPH3 expression and different chemotherapy regimens after surgery

Chemotherapy regimen	GOLPH3 expression	Total (n)	Recurrence (n)	Non-Recurrence (n)	P-value
Anthracycline + cyclophosphamide sequential taxane (n=74)	GOLPH3 +	31	7	24	0.035
	GOLPH3 ++/+++	43	20	23	
5-fluorouracil + anthracycline + cyclophosphamide (n=79)	GOLPH3 +	51	5	46	0.010
	GOLPH3 ++/+++	28	12	16	
Taxane + cyclophosphamide (n=30)	GOLPH3 +	25	1	24	0.190
	GOLPH3 ++/+++	5	1	4	
Others (n=33)	GOLPH3 +	6	0	6	0.027
	GOLPH3 ++/+++	27	14	13	

## DISCUSSION

In this study, the clinical significance of GOLPH3 in breast cancer was explored. GOLPH3 was found to be highly expressed in breast cancer tissue and cell lines. GOLPH3 silencing inhibited cell migration, invasion, and proliferation, and promoted apoptosis of breast cancer cells. Importantly, patients with GOLPH3 overexpression had poor DFS and OS, and GOLPH3 expression was associated with clinicopathologic factors such as age, TNM classification, and molecular subtype but did not correlate with surgery type. Furthermore, patients with high GOLPH3 expression showed resistance to chemotherapy.

Compared with adjacent noncancerous tissue and fibroadenoma samples, GOLPH3 was significantly upregulated in breast cancer tissue. This finding is in accordance with a previous report [26]. Interestingly, GOLPH3 is also elevated in lung [18][23], prostate [21], ovarian epithelial [20], hepatoma [22], and rectal cancers [25], implying that GOLPH3 plays a role in the occurrence or development of tumors and may have potential for use in cancer diagnosis. A malignant tumor is regarded as a neoplasm with aggressive, migration and rare apoptosis [30]. The migration and invasion of cancer cells comprise a complex multi-step process including reorganization of the cytoskeleton, degradation of the extracellular matrix, adhesion, and de-adhesion [27–29]. In this study, downregulation of GOLPH3 siRNA not only significantly reduced the migration, invasion, and proliferation of two breast cancer cell lines but also promoted cell apoptosis, indicating that GOLPH3 affects breast cancer development. Similar to breast cancer, GOLPH3 also plays a role in other cancers such as lung [18,23], bladder [19], and renal cell cancers [25]. Thus, GOLPH3 is not merely a diagnostic marker for breast cancer but may also have potential as a significant therapeutic target just like trastuzumab target human epidermal growth factor receptor 2.

Our investigation also revealed that high GOLPH3 expression affected the DFS and OS in breast cancer patients. Similar results were found for renal cell carcinoma [24,25]. Xue *et al.* [24] noticed that high GOLPH3 expression correlated with poor OS in 218 renal cell carcinoma patients [24], and Zhu *et al.* [25] found that GOLPH3 overexpression correlated with a worse DFS and OS in 77 rectal carcinoma patients compared with 70 patients with low levels of GOLPH3 expression. Meanwhile, Zeng *et al.* [26] reported that the overexpression of GOLPH3 was associated with poor OS in breast cancer. Our results not only confirmed that patients with high GOLPH3 expression had worse OS but also revealed that high GOLPH3 expression correlated with worse DFS in breast cancer.

We presented a systematic study of the correlation between GOLPH3 and clinical pathological characteristics including age, TNM classification, and molecular subtype. This is the first study to evaluate how GOLPH3 expression influences the survival of breast cancer patients in different age groups. We demonstrated that the risk of recurrence increases with increasing age in patients with high GOLPH3 expression; thus, GOLPH3 may be a suitable predictor of the survival of breast cancer patients >40 years of age. We also performed a comprehensive investigation between GOLPH3 and TNM classification and found that high GOLPH3 expression led to a worse prognosis in breast cancer patients with advanced tumor invasion depth and lymphatic metastasis. Xue *et al.* [24] investigated the association of GOLPH3 with tumor invasion depth in renal cell carcinoma and showed that poor OS was observed in the high GOLPH3 expression group. Similar results were observed in our study in patients with high GOLPH3 expression with advanced lymphatic metastasis. However, Xue *et al.* [24] did not show the correlation between GOLPH3 and other clinical features such as age, molecular subtype, or surgery type. Importantly, we found that high GOLPH3 expression led to a poor DFS and OS in all molecular subtypes, suggesting that GOLPH3 may be an important therapeutic target in breast cancer. In addition, surgery type had no influence on the survival of patients with high GOLPH3 expression.

Systemic chemotherapy, including neoadjuvant chemotherapy and adjuvant chemotherapy, is a very important treatment strategy for breast cancer patients [31–37]. To date, no studies have investigated the association between GOLPH3 expression and neoadjuvant chemotherapy in breast cancer. Our study showed that in breast cancer patients, GOLPH3 overexpression was associated with resistance to neoadjuvant chemotherapy, especially to the most frequently used regimen of anthracycline+taxane+cyclophosphamide, although these results were based on data from a small number of patients. This result is in agreement with a previous report that showed that GOLPH3 overexpression leads to failure of neoadjuvant chemotherapy in locally advanced rectal cancer [25]. For patients who received adjuvant chemotherapies after surgery, our results demonstrated that patients with high GOLPH3 expression had a higher risk of disease recurrence and death if the chemotherapy regimen was the anthracycline+cyclophosphamide sequential taxane regimen or 5-fluorouracil+anthracycline+cyclophosphamide. However, in patients who received the taxane+cyclophosphamide regimen, GOLPH3 expression did not affect DFS and OS. For patients who received the taxane+cyclophosphamide regimen, their disease was not so severe that GOLPH3 expression impacted survival. Together, these data suggest that GOLPH3 overexpression may attenuate the effects of anthracycline or 5-fluorouracil. Interestingly, it has been reported that high GOLPH3 expression is associated with favorable prognosis in colorectal cancer patients treated with 5-fluorouracil adjuvant chemotherapy [38]. This contradictory result may be attributed to differences between breast cancer and colorectal cancer.

In summary, our results suggest that GOLPH3 is a very important oncogene that plays a significant role in breast cancer. High GOLPH3 expression usually suggests poor survival and resistance to chemotherapy in breast cancer. Therefore, GOLPH3 has high prospective value in diagnosis and may serve as a clinical indicator and prognostic marker for patients. However, due to the limitations of the current study, further investigations on the clinical use of GOLPH3 are required to confirm these data.

## MATERIALS AND METHODS

### Patient information, follow up, and tissue samples

A total of 249 breast cancer tissue samples, 72 adjacent noncancerous tissue (ANT), and 20 fibroadenoma samples were obtained from patients who underwent surgical treatment in Tumor Hospital of Guangxi Medical University from 2007 to 2013. We separated the tissue samples into two parts: one part was immediately frozen and stored at −80°C in the Tumor Tissue Bank of Tumor Hospital of Guangxi Medical University for gene expression analysis, and the other part was fixed in formalin and embedded in paraffin for immunohistochemical analysis. All slides were blindly reviewed by two pathologists and a consensus diagnosis was reached. Pathologic classification and stage was determined according to the American Joint Committee on Cancer criteria. Molecular subtypes were determined according to the 2013 St Gallen criteria. Patients were followed up from the time of surgery to death or last contact. The protocols of the study were approved by Ethnics Committees of Tumor Hospital of Guangxi Medical University.

### Cell culture, siRNA for GOLPH3, and transfection

The breast cancer cell lines (MDA-MB-231 and MCF-7) were purchased from Cell Bank of the Chinese Academy of Science (Shanghai, China). The cells were cultured in Dulbecco's Modified Eagle Medium with 15% fetal bovine serum, 0.5% streptomycin, and 0.5% penicillin (Life Technologies, Washington DC, USA) at 37°C and 5% CO2. GOLPH3 siRNA was designed by Gene Pharma (Shanghai, China) to knockdown GOLPH3 expression. The sequence of siRNA was 5′-GUUA AGAAAUGUACGGGAATT-3′. A scrambled siRNA (scram-siRNA) was used as a negative control. The sequence of the scram-siRNA was 5′-UUCUC CGAACGUGUCACGUTT-3′. The breast cancer cell lines were transfected with either GOLPH3-siRNA or scram-siRNA according to the manufacturer's protocol. Forty-eight hours after transfection, qPCR assays and Western blotting analyses were performed to analyze GOLPH3 mRNA and protein expression.

### Immunohistochemistry

GOLPH3 antibodies (1:150 dilution; ab91492, Abcam, Cambridge, MA, USA) were chosen for immunohistochemistry. Sections were incubated for 1 h at 60°C, dewaxed for 40 min in dimethylbenzene, and rehydrated. After washing in phosphate-buffered saline (PBS) solution, the sections were blocked in 5% normal goat serum for 30 min. To inhibit endogenous peroxidase activity, the sections were treated with 0.5% hydrogen peroxide methanol for 30 min. Primary antibodies were added dropwise to the sections, after which the sections were incubated overnight at 4°C. Next, sections were rewarmed for 30 min at room temperature, washed three times with PBS for 5 min, and incubated with the appropriate secondary antibodies (1:200 dilution; ab186696, Abcam) for 30 min at 37°C. This was followed by another three 5-min wash with PBS, after which 0.05% DAB was added for visualization of immunoreactivity. Sections were dehydrated in a series of 100%, 95%, 80%, and 75% graded ethanol and counterstained with hematoxylin. GOLPH3 expression was assessed by evaluating the proportion and intensity of positively stained carcinoma cells. A score was assigned to represent the estimated percentage of positively stained carcinoma cells as follows: 0: none, 1: ≤ 50%, 2: 50–75%, and 3: ≥ 75%. An intensity score was assigned to represent the average estimated intensity of staining in positive carcinoma cells as follows: 0: none, 1: weak, 2: intermediate, and 3: strong. The proportion score and intensity score were multiplied to obtain a total score ranging from 0 to 9. The immunohistochemistry results were classified based on total scores with 0–4 classified as +, 5–6 indicating ++, and 7–9 indicating +++ [19].

### Western blotting

Protein was extracted by RIPA buffer (BOSTER, Wuhan, China) containing 1mM/L PMSF (BOSTER, Wuhan, China). The concentration of total protein in each sample was measured by the BCA protein assay kit (BOSTER, Wuhan, China), after which 10% SDS-PAGE gels were used to separate 50 μg protein. Then the proteins were electrophoretically transferred to polyvinylidene fluoride membranes (Millipore, MA, USA). After blocking in Tris-buffered saline with Tween 20 (10 mM/L Tris-HCL pH 7.5, 150 mM/L NaCl, and 0.05% Tween-20) containing 5% non-fat milk for 1 h, the membranes were incubated overnight at 4°C with primary anti-GOLPH3 monoclonal antibody (1:1000 dilution; ab91492, Abcam) and α-tubulin (1:6000 dilution; ab7291, Abcam). Then the membranes were washed three times with TBST for 10 min and probed with secondary antibody (1:10000 dilution, ab186696, Abcam) for 1 h at room temperature. Signals were visualized with enhanced chemiluminescence solution (BOSTER) by exposure to film.

### RNA extraction, RT-PCR, and qPCR

Total RNA from both cell lines and tissue samples was extracted using the TaKaRa MiniBEST Universal RNA Extraction Kit (TaKaRa, Shiga, Japan) according to the manufacturer's instructions. The RNeasy Mini Kit (TaKaRa) was used for further purification. cDNA was both reverse transcribed using the PrimeScript™RT reagent Kit (TaKaRa), after which the cDNA underwent a 42°C 2 min reaction. Then 10 μL DNA erase compound was mixed with PrimeScript RT Enzyme Mix I, RT Primer Mix, 5× PrimeScript Buffer 2 (for Real Time) and RNase Free dH_2_O in a 20 μL reaction. Then a PCR reaction at 37°C for 15 min, and at 85°C 5 s was performed. The cDNA was preserved at 4°C for qPCR. GOLPH3 forward and reverse primers were synthesized. The GOLPH3 primers were as follows: 5′- GGGCGACT CCAAGGAAAC −3′ for forward and 5′- CAGCCACGTAA TCCAGATGAT −3′ for reverse primers. GAPDH was used as the internal control with specific primers: 5′-GCACCGTCAAGGCTGAGAAC-3′ for forward and 5′-TGGTGAAGACGCCAGTGGA-3′ for reverse. The reaction system contained SYBR® Premix Ex Taq II (Tli RNaseH Plus, PCR forward primer, PCR reverse primer, ROX Reference Dye (50×), dH_2_O, and cDNA templates. Gene and species-specific primer/internal reference were performed on the Applied Biosystems StepOne Plus Real-time PCR system at 95°C for 30 s for 1 cycle and at 95°C for 3 s and then at 60°C for 30 s for 40 cycles. The data were analyzed using the comparative threshold cycle (2^−ΔΔCT^) method.

### Migration and invasion assay

A total of 2×10^5^ MCF-7 cells/well and 1×10^4^ MDA-MB-231 cells/well were resuspended in culture medium without serum and seeded. For the migration assay, transfected and control cells were plated in the upper chamber without matrigel. The same concentration of cells was plated on an 8 μm pore coated with 60 μL matrigel for the invasion assay. Culture medium that contained 10% serum was used in the lower chamber. After 24 h, cells that did not invade or migrate were removed. The migrated and invaded cells were fixed in 4% polysorbate, dyed with the Giemsa stain, quantified, and photographed.

### CCK-8 assay

Cell proliferation was analyzed by the CCK-8 assay. Briefly, 5×10^4^ cells/mL and 100 μL suspension were added to a 96-well plate. Cells were continuously cultured at 37°C for 4 h. Then 10 μL CCK-8 solution (BestBio, Shanghai, China) was added to each well at 24, 48, and 72 h after culturing followed by incubation at 37°C for 4 h. The optical density of the suspensions was determined by absorbance at 450 nm.

### Flow cytometer analysis

Flow cytometry was used for cell apoptosis analysis. Cells were harvested 48 h after transfection, and washed twice with ice-cold PBS. 1× staining buffer was mixed to adjust the cell concentration to 1×10^6^ cells/mL. Then 400 μL of 1× binding buffer was mixed and 100 μL cell suspension was incubated with 3 μL propidium iodide (PI) and 5 μL Annexin V-FITC for 15 min in the dark. For cell cycle analysis, cells were fixed in 70% ethanol and stored at −20°C overnight. The suspension was stained with PI at 4°C in the dark. The apoptosis and cell cycle of cells were analyzed with the BD Accuri C6 flow cytometry system (BD Biosciences, San Jose, CA, USA).

### Chemotherapy treatment

A total of 54 patients in the cohort of 249 patients received neoadjuvant chemotherapy before surgery. Patients who received neoadjuvant chemotherapy strategies were classified into three subgroups. The first subgroup comprised 22 patients who received the anthracycline+taxane+cyclophosphamide regimen. Of these 22 patients, 12 underwent two to four cycles of the TAC regimen (i.e., docetaxel 75 mg/m^2^, pirarubicin 60 mg/m^2^, and cyclophosphamide 600 mg/m^2^ iv on day 1, every 3 weeks). The remaining 10 patients received two to four cycles of the TEC regimen (docetaxel 75 mg/m^2^, epirubicin 100 mg/m^2^, and cyclophosphamide 600 mg/m^2^ iv on day 1, every 3 weeks). The second subgroup comprised 20 patients who received two to five cycles of the 5-fluorouracil+anthracycline+cyclophosphamide regimen. Of these 20 patients, 7 received two to four cycles of the FAC regimen (i.e., 5-fluorouracil 600 mg/m^2^, pirarubicin 60 mg/m^2^, and cyclophosphamide 600 mg/m^2^ iv on day 1, every 3 weeks). The remaining 13 patients received two to five cycles of the FEC regimen (i.e., 5-fluorouracil 600 mg/m^2^, epirubicin 100 mg/m^2^, and cyclophosphamide 600 mg/m^2^ iv on day 1, every 3 weeks) The third subgroup contained 12 patients who received other neoadjuvant chemotherapy regimens. A total of 216 patients received adjuvant chemotherapy after surgery. We divided the patients into four treatment groups: (1) 74 patients were treated with anthracycline+cyclophosphamide sequential taxane; 55 patients received AC→T (4 cycles of pirarubicin 60 mg/m^2^ iv on day 1, cyclophosphamide 600 mg/m^2^ iv on day 1, every 2 or 3 weeks→4 cycles of docetaxel 75 mg/m^2^ iv on day 1, every 2 or 3 weeks). 19 patients received EC→T (four cycles of epirubicin 100 mg/m^2^ iv on day 1, cyclophosphamide 600mg/m^2^ iv on day 1, every 2 or 3 weeks→four cycles of docetaxel 75 mg/m^2^ iv on day 1, every 2 or 3 weeks). (2) 79 patients received the 5-fluorouracil+anthracycline+cyclophosphamide regimen, as outlined above. (3) 30 patients underwent four to six cycles of the taxane+cyclophosphamide regimen (docetaxel 75 mg/m^2^ iv on day 1, cyclophosphamide 600 mg/m^2^ iv on day 1, every 3 weeks). (4) 33 patients received other adjuvant chemotherapy regimens.

### Statistical analysis

All statistical analyses were performed with SPSS Statistics 21.0 (IBM, Chicago, IL, USA) and Graphpad Prism 6.0. Comparisons in continuous outcomes between groups for statistical significance were carried out with a two-tailed paired Student *t*-test. The relationship between GOLPH3 expression and clinicopathological characteristics was evaluated by the chi-squared test, and the Fisher's exact test was used when the unit was less than six. OS and DFS were assessed by the Kaplan-Meier method and compared by the log-rank test. COX regression models were used for survival data. P values less than 0.05 were considered statistically significant. ^*^P < 0.05, ^**^P <0.01, ^***^P < 0.001, relative to the control.

## SUPPLEMENTARY FIGURES AND TABLE


