# Supplementary material for: GOLPH3: a novel biomarker that correlates with poor survival and resistance to chemotherapy in breast cancer
Source: Oncotarget. 2017 Oct 19;8(62):105155–69. doi: 10.18632/oncotarget.21927 (PMC5739628; doi:10.18632/oncotarget.21927)
Supplement: Supplementary file 1 [file oncotarget-08-105155-s001.pdf]

GOLPH3: a novel biomarker that correlates with poor survival and resistance to chemotherapy in breast cancer

SUPPLEMENTARY MATERIALS

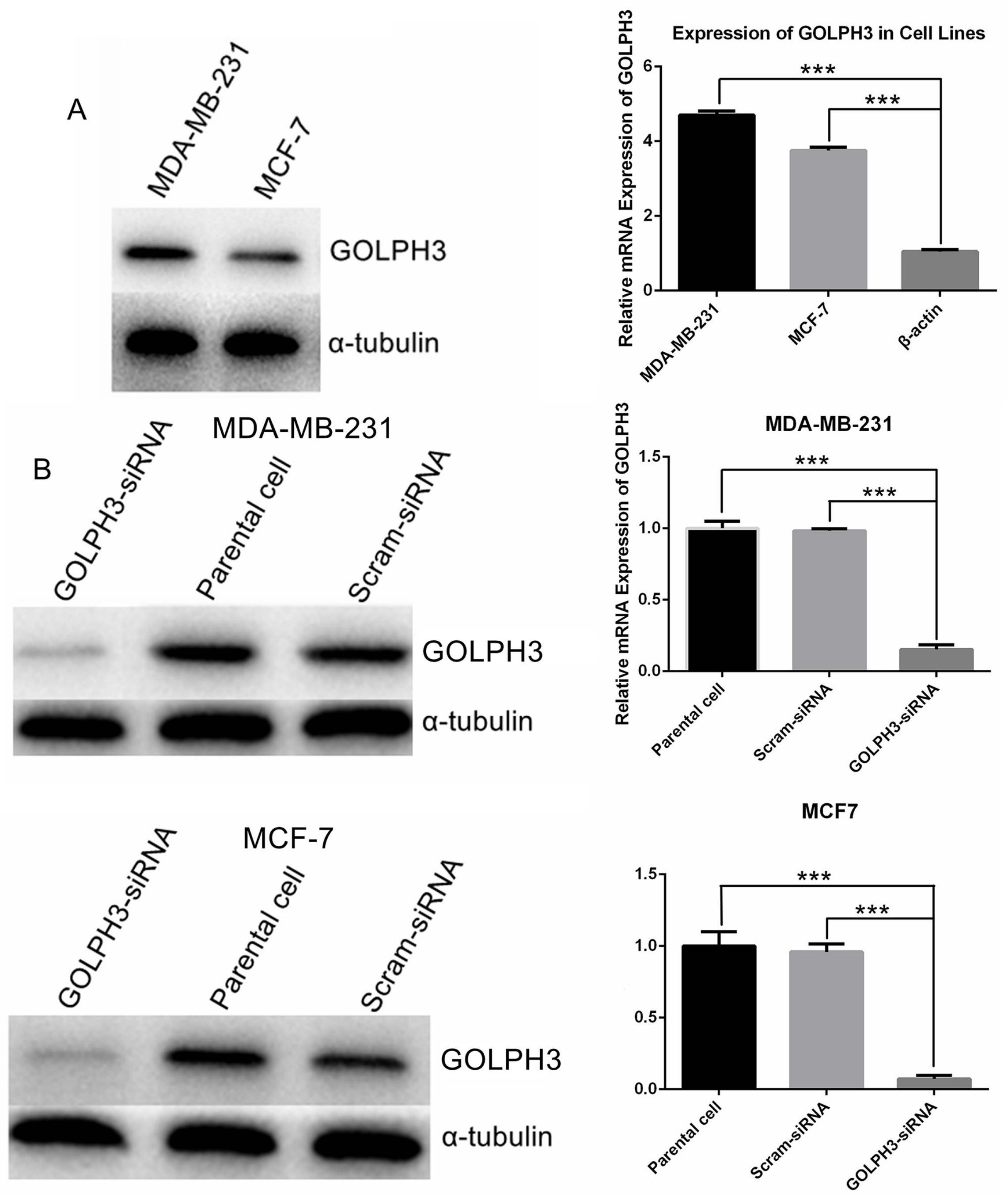

Supplementary Figure 1: Expression of GOLPH3 in cell lines. (A) GOLPH3 expression in breast cancer MDA-MB-231 and MCF-7 cells was analyzed by Western blotting and real-time PCR. **(B)** Effects of GOLPH3 silencing were analyzed by Western blotting and real-time PCR. All values are the mean ±SD of three independent experiments. ***, *p*<0.001.

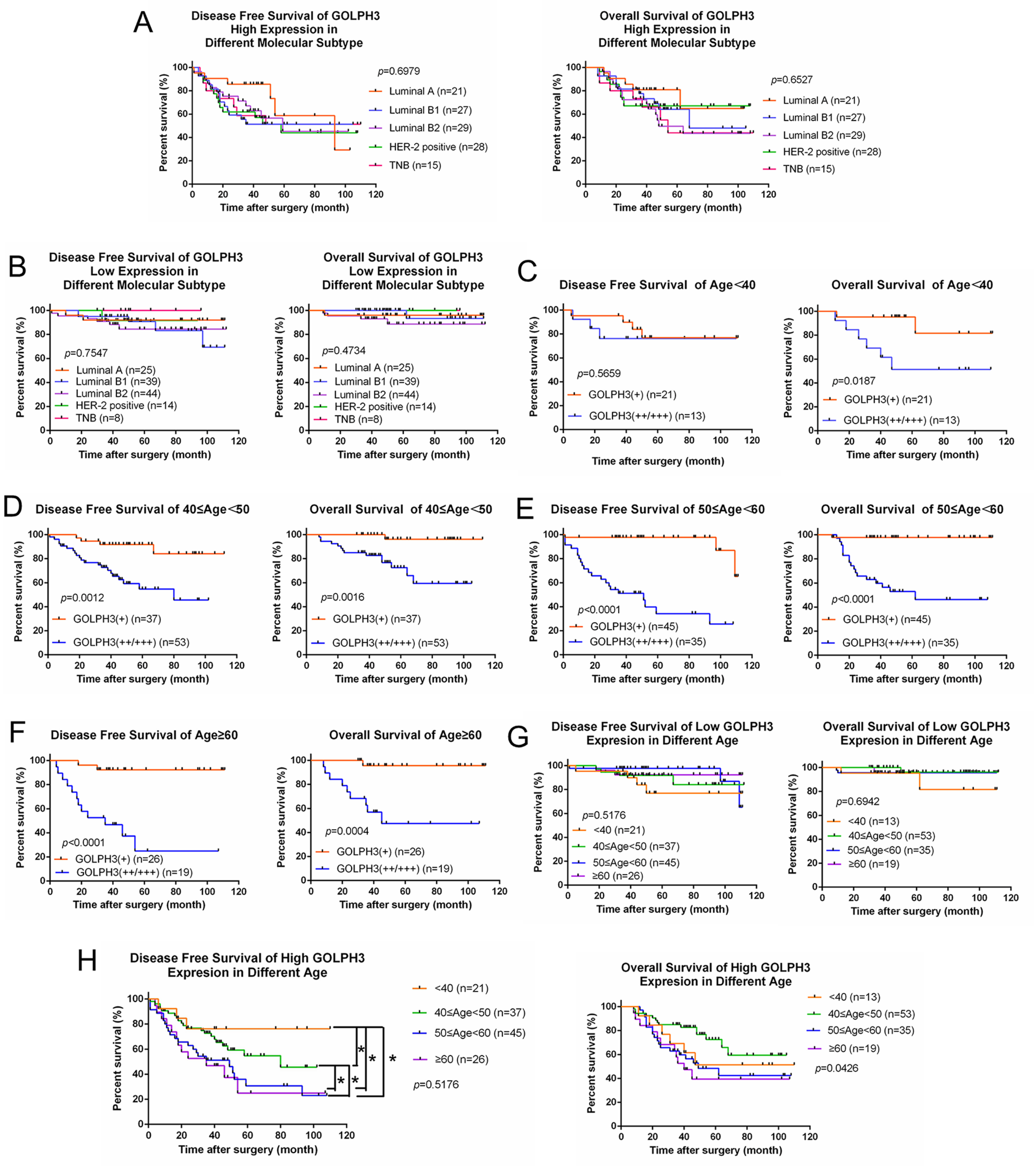

Supplementary Figure 2: DFS and OS of breast cancer patients with different molecular subtypes and age. (A) DFS and OS of patients with high GOLPH3 expression in different molecular subtypes. **(B)** DFS and OS of patients with low GOLPH3 expression in different molecular subtypes. **(C)** DFS and OS of patients with age<40. **(D)** DFS and OS of patients with 40≤Age<50. **(E)** DFS and OS of patients with 50≤Age<60. **(F)** DFS and OS of patients with Age≥60. **(G)** DFS and OS of patients with low GOLPH3 expression in different age groups. **(H)** DFS and OS of patients with high GOLPH3 expression in different age.*, $p<0.05$.

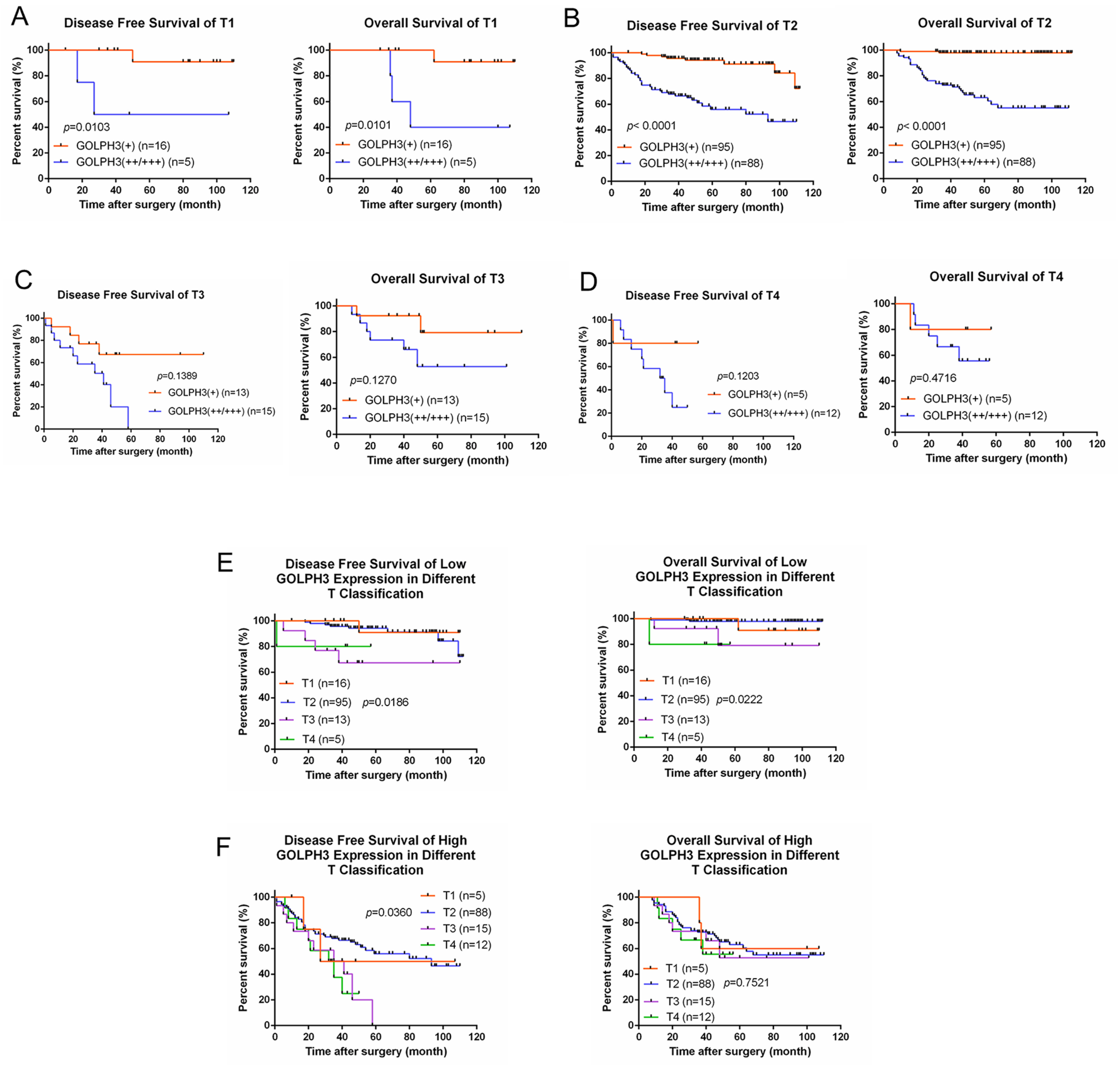

Supplementary Figure 3: DFS and OS of breast cancer patients with different T classifications. **(A)** DFS and OS of patients with T1 lesions. **(B)** DFS and OS of patients with T2 lesions. **(C)** DFS and OS of patients with T3 lesions. **(D)** DFS and OS of patients with T4 lesions. **(E)** DFS and OS of patients with low GOLPH3 expression and different T classifications. **(F)** DFS and OS of patients with high GOLPH3 expression and different T classifications.

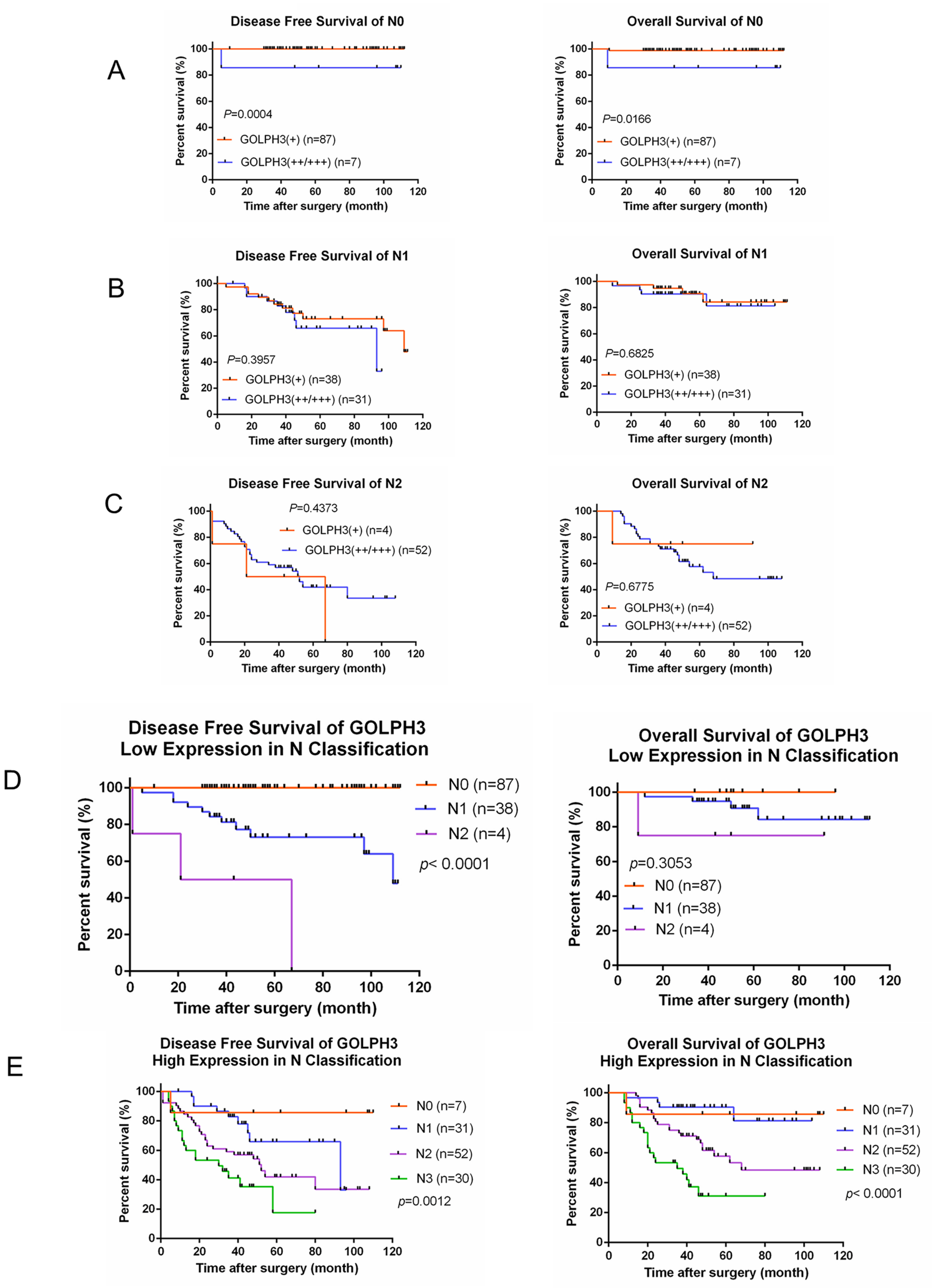

Supplementary Figure 4: DFS and OS of breast cancer patients with different N classifications. **(A)** DFS and OS of patients with N0. **(B)** DFS and OS of patients with N1. **(C)** DFS and OS of patients with N2. **(D)** DFS and OS of patients with low GOLPH3 expression in different N classification. **(E)** DFS and OS of patients with high GOLPH3 expression in different N classification.

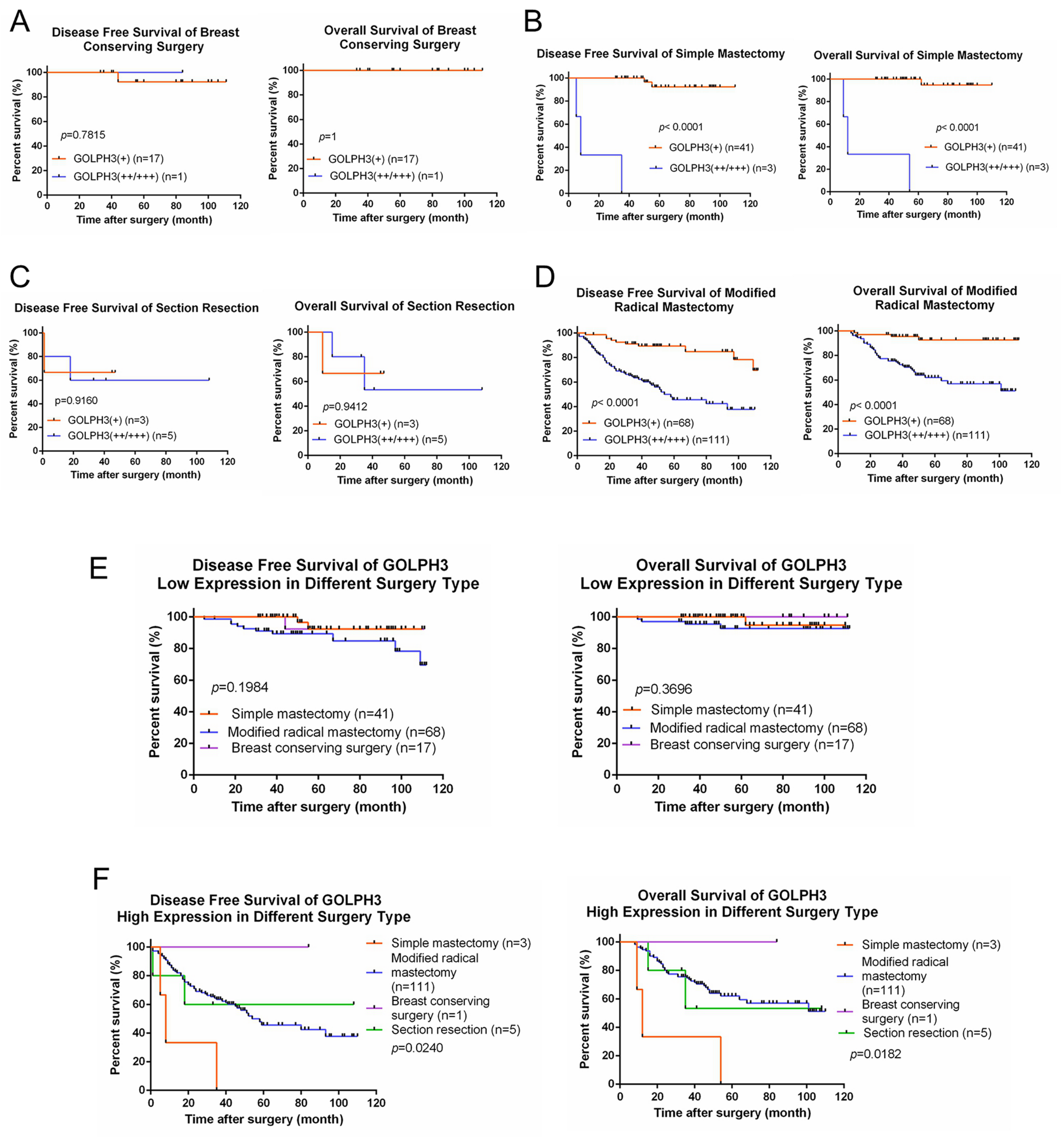

Supplementary Figure 5: DFS and OS of breast cancer patients with different surgery types. **(A)** DFS and OS of patients received breast-conserving surgery. **(B)** DFS and OS of patients who received simple mastectomy. **(C)** DFS and OS of patients who received modified radical mastectomy. **(D)** DFS and OS of patients with low GOLPH3 expression and different surgery types. **(E)** DFS and OS of patients with low GOLPH3 expression and different surgery types. **(F)** DFS and OS of patients with high GOLPH3 expression and different surgery types.

Supplementary Table 1: Items comparation with GOLPH3 high expression patients in every clinicopathological feature

Variables	Items comparation	X^2 value	P-value
**Age (①:<40 ②:40≤Age<50 ③:51≤Age<60 ④:≥60)**	①VS②	4.228	0.046
	①VS③	0.298	0.680
	①VS④	0.128	0.818
	②VS③	3.888	0.065
	②VS④	3.348	0.099
	③VS④	0.027	1.000
**T classification (①:T1②:T2③:T3④:T4)**	①VS②	4.476	0.039
	①VS③	4.400	0.045
	①VS④	8.315	0.008
	②VS③	0.292	0.686
	②VS④	3.150	0.126
	③VS④	1.276	0.351
**N classification (①:N0②:N1③:N2④:N3)**	①VS②	31.267	0.000
	①VS③	107.284	0.000
	①VS④	93.053	0.000
	②VS③	31.829	0.000
	②VS④	26.814	0.000
	③VS④	2.247	0.293
**M classification (①:M0②:M1)**	①VS②	12.372	0.000
**Molecular subtype (①Luminal A ②Luminal B1 ③Luminal B2 ④Her-2 positive ⑤Triple negative)**	①VS②	0.249	0.699
	①VS③	0.407	0.570
	①VS④	4.517	0.051
	①VS⑤	2.352	0.201
	②VS③	0.020	1.000
	②VS④	7.591	0.009
	②VS⑤	4.044	0.055
	③VS④	8.570	0.006
	③VS⑤	4.578	0.054
	④VS⑤	0.063	1.000
**Surgery type (①: Simple mastectomy ②: Modified radical mastectomy ③: Breast conserving surgery ④: Section resection)**	①VS②	43.057	0.000
	①VS③	0.034	1.000
	①VS④	16.122	0.001
	②VS③	21.250	0.000
	②VS④	0.001	1.000
	③VS④	10.117	0.004

Note: Luminal A: ER (+) and PR (+), PR≥20%, Her-2 (-), Ki-67<14%; Luminal B1: ER (+), Her-2 (-), Ki-67≥14% or PR<20%; Luminal B2: ER (+), Her-2 (+), Ki-67≥14% or PR<20%; Her-2 positive: ER (-) and PR (-), Her-2 (+); Triple negative: ER (-) and PR (-), Her-2 (-).The results was corrected to three decimal places.
